# Aligned PLGA/nHA fiber scaffolds for enhancing osteogenic differentiation of human periodontal ligament stem cells

**DOI:** 10.3389/fbioe.2026.1802077

**Published:** 2026-06-11

**Authors:** Jiaying Zhai, Xiaohuan Peng, Yuanyuan Hu, Zhiyuan Li, Wenxiu Li, Chengguang Zhu, Li Zheng, Jin Chen, Yajing Wang

**Affiliations:** 1 The Affiliated Stomatological Hospital of Guizhou Medical University, Guizhou Medical University, Guiyang, China; 2 The Stomatology of Guizhou Medical University, Guizhou Medical University, Guiyang, China; 3 Department of Stomatology, People’s Hospital of Dafang, Bijie, Guizhou, China; 4 Key Laboratory of Biology and Medical Engineering, School of Biology and Engineering, Guizhou Medical University, Guiyang, China

**Keywords:** aligned fiber scaffolds, alveolar bone regeneration, hPDLSCs, micro-/nanostructures, nano-hydroxyapatite

## Abstract

**Object:**

The aim of this study was to investigate the effects of aligned poly (lactic-co-glycolic acid)/nano-hydroxyapatite (PLGA/nHA) fiber scaffolds with micro-/nanostructures on the osteogenic differentiation potential of human periodontal ligament stem cells (hPDLSCs).

**Methods:**

Aligned/random PLGA and PLGA/nHA fiber scaffolds with micro-/nanostructures were created by electrospinning. Comprehensive characterization of the scaffolds with scanning electron microscopy (SEM), X-ray diffraction, Fourier transform infrared spectroscopy, mechanical testing, thermogravimetric analysis and hydrophilicity was performed. In addition, they were observed if they could promote the osteogenic differentiation of hPDLSCs by RT-qPCR, SEM, alkaline phosphatase staining and alizarin red staining. The subcellular distribution of YAP protein between the nucleus and cytoplasm was detected by YAP immunofluorescence staining.

**Results:**

Aligned PLGA/nHA fiber scaffolds had a better mechanical strength, thermal stability, hydrophilicity than random fiber scaffolds. Furthermore, aligned PLGA/nHA scaffolds also promoted cell adhesion, obvious cytoskeletal elongation and enhanced osteogenic differentiation of hPDLSCs was observed by the upregulation of the key genes (OCN, RUNX2, OPN) expression, increased the activity of alkaline phosphatase, and positive for alizarin red. Meanwhile, the a-PLGA/nHA scaffolds showed the strongest nuclear YAP fluorescence intensity.

**Conclusion:**

The synergistic effect of fiber arrangement and nHA can be used to increase the osteogenic differentiation of hPDLSCs and the composite scaffold developed may have translational potential for alveolar bone regeneration.

## 1 Introduction

As a chronic inflammatory disorder, periodontitis drives periodontal pocket development and alveolar bone destruction, representing the foremost cause of adult dentition loss (Shaddox et al., 2021; Kapila, 2021; Genco and Sanz, 2020). The ultimate goal of periodontal therapy is the regeneration of lost tissues (Santos et al., 2023), among which the reconstruction of the missing alveolar bone is of paramount importance. Nonetheless, due to the structural complexity of periodontal tissues, conventional non-surgical therapies (e.g., supragingival scaling, subgingival curettage, and root planing) can only alleviate the progression of periodontitis to a limited extent. Moreover, surgical therapies (e.g., guided tissue regeneration) are often uncertain and fail to achieve complete restoration of periodontal tissues, particularly the alveolar bone. With the development of tissue engineering, engineered scaffolds are increasingly tailored to emulate the physicochemical and biological properties of periodontal tissues and have demonstrated promising regenerative efficacy (He et al., 2021; Cui et al., 2021). This is a potential therapeutic approach for the regeneration of alveolar bone.

In addition to mechanical qualities, aligned architecture has been recognized as a critical biological and physical determinant of bone regeneration efficiency (Soheilmoghaddam et al., 2020). Compared with randomly oriented scaffolds, aligned scaffolds efficiently direct cells to align along the fiber long axis and markedly enhance cell migration and proliferation (Russo et al., 2020; Puhl et al., 2020; El Khatib et al., 2020). Additionally, they can promote the differentiation of adult stem cells and enhance the cellular reprogramming of induced pluripotent stem cells (Russo et al., 2020; Puhl et al., 2020; El Khatib et al., 2020). Among them, ordered scaffolds with micro/nano-structures have an excellent ability to promote the osteogenic differentiation of stem cells and are expected to be alternative materials for repairing various bone tissue defects in clinical practice (Xie et al., 2021; Xiong et al., 2022). The underlying mechanism lies in the fact that scaffolds with micro-/nanostructures possess favorable mechanical stiffness and bulk modulus, enabling more accurate recapitulation of the extracellular matrix (ECM) microenvironment (Verdugo-Avello et al., 2025). The micro-/nano-topographical features of the scaffold surface actively direct the reorganization and remodeling of the cytoskeletal network, thereby preserving tensile homeostasis of cells within a three-dimensional space (Yang et al., 2025; Chen et al., 2021). Therefore, it is necessary to create aligned scaffolds with micro-/nanostructures to optimize the tissue engineering scaffolds. Currently, aligned micro/nano multiscale scaffolds have been extensively employed in tissue regeneration (e.g., bone (Nevado et al., 2020) and muscle (Zhang et al., 2020; Yeo and Kim, 2019). However, their application to periodontal tissues (particularly to alveolar bone) remains comparatively underexplored. Indeed, one major challenge stems from the intricate architecture of the alveolar bone. The native alveolar bone matrix exhibits a unique micro-/nanostructure characterized by an aligned type I collagen scaffold and the uniform interfibrillar deposition of nanoscale hydroxyapatite crystals (Moten et al., 2022). This structure provides the necessary mechanical competence while also presenting a complex set of physical and biochemical signals. These signals thereby orchestrate cellular adhesion, migration, and differentiation, which are critical for driving the process of bone tissue regeneration (Shi et al., 2024; Shi et al., 2021). An aligned scaffold has been demonstrated to be capable of inducing cementogenic differentiation and tissue formation (Zhang et al., 2025), Moreover, scaffolds that simulate key features of the periodontal microstructure can achieve spatiotemporal delivery of bioactive cues through their design. This targeted delivery thereby promotes a regeneration of the periodontal complex (Hua et al., 2023). Consequently, the fabrication of aligned multiscale scaffolds integrating biocompatible organic and inorganic phases represents a promising strategy for alveolar bone regeneration.

PLGA is used to repair bone defects, to release drugs and for periodontal regeneration due to being biodegradable and adjustable degradation rates (Gentile et al., 2014; Zhao et al., 2021). Adding nanoscale ceramic particles can also enhance its mechanical strength and biocompatibility (Chen et al., 2024; Wang et al., 2021), and mitigate the inherent shortfalls of low load-bearing capacity, poor cellular affinity and continuous inflammatory stimulation of PLGA (Lauritano et al., 2020), significantly boosting the therapeutic effect. Among many of the bioceramics, HA is a biologically active, non-toxic, and it has intrinsic osteoinductive and osteoconductive. It is often added as an active component or reinforcing phase to natural or synthetic polymer matrices to make bone tissue engineering scaffolds with good performance (Cakmak et al., 2020; Zaszczyńska et al., 2024; Mirică et al., 2021). A large number of experiments have confirmed that PLGA/HA composite scaffolds can significantly improve cell proliferation, migration and differentiation, thus verifying their osteogenic capacity in bone tissue engineering (Hamiti et al., 2025; Wang et al., 2024; Tong et al., 2025). Notably, the propensity of nHA to agglomerate within the polymer matrix creates stress-concentration sites that compromise material toughness and restrict its clinical advancement in orthopedics (Deng et al., 2020; Tang et al., 2024). Existing research is often limited to systems featuring either random nHA distributions or disordered fibers, failing to simultaneously incorporate aligned fiber guidance, micro-/nanostructures, and nHA to mimic the native bone matrix (Shi et al., 2021), thus constraining their regenerative outcomes in alveolar bone applications.

In this work, aligned PLGA/nHA scaffolds with micro-/nanostructures were fabricated based on our previous work (Wang et al., 2015). Briefly, nano-hydroxyapatite (nHA) was initially prepared by the wet chemical precipitation method, and sodium polyacrylate (PAAS) was added to enhance the dispersion of nHA in the polymer matrix. Subsequently, aligned PLGA/nHA composite scaffolds with micro-/nanostructures were prepared through electrospinning by changing the rotation speed of the collector to partially mimic the microstructure of alveolar bone. After comprehensive physicochemical characterization of the composite scaffolds, the proliferation, adhesion and osteogenic differentiation of human periodontal ligament stem cells (hPDLSCs) in the composite scaffolds will be further studied. The aim of this study was to preliminarily reveal the impact of aligned composite scaffolds with micro-/nanostructures on the osteogenic differentiation of hPDLSCs and provide references for preparing bioactive implantable materials for alveolar bone regeneration.

## 2 Materials and methods

### 2.1 Materials

The PLGA (LA: GA = 75:25, M_W_ = 76,000–111,500 g/mol), 1,1,1,3,3,3-hexafluoro-2-propanol (HFIP), and PAAS were obtained from Aladdin Co., Ltd. (Shanghai, China). The α-MEM culture medium was purchased from Gibco (Thermo Fisher Scientific Inc., Waltham, MA, United States). The fetal bovine serum and alizarin red staining solution were purchased from Cyagen Biosciences Inc. (Guangzhou, China). The CCK-8 assay kit was obtained from Dojindo Molecular Technologies, Inc., Japan. The 2.5% glutaraldehyde and phalloidin were acquired from Solarbio Science and Technology Co., Ltd. (Beijing, China). The 4′,6-diamidino-2-phenylindole (DAPI) and the BCIP/NBT alkaline phosphatase color development kit, live/death staining kit, and calcium ion detection kit were purchased from Beyotime Biotechnology Co., Ltd. (Shanghai, China). TRIzol reagent was obtained from Takara Bio Inc. (Kusatsu, Shiga, Japan). The qPCR SYBR Green Master Mix and HiScript HI RT SuperMix for qPCR kit were purchased from Vazyme Biotech Co., Ltd. (Nanjing, China). The primary antibody against YAP (ET1608-30) was purchased from Huaan Biotechnology (Wuhan, China). The secondary antibody (Cy3-conjugated goat anti-rabbit, GB21303) was obtained from Servicebio (Wuhan, China). Both antibodies were used at the dilutions recommended by the manufacturers.

### 2.2 Preparation of nHA

Initially, a solution of (NH_4_)_2_HPO_4_ (0.133 M, 125 mL) was added dropwise to a solution of Ca(NO_3_)_2_·4H_2_O (0.2 M, 125 mL). Subsequently, we incorporated 0.5 mL of 50% PAAS into the solution. The mixture (pH = 10) was heated at 80 °C for 8 h. Following the reaction, the product was subjected to multiple washings, centrifugation, and freeze-drying to yield nHA powders.

### 2.3 Preparation of PLGA/nHA fiber scaffolds

Briefly, PLGA (1.6 g) was solubilized in 10 mL HFIP and magnetically stirred for 4 h to obtain a 16% (w/v) PLGA solution. The nHA (0.144 g) powder was incorporated into 10 mL HFIP and sonicated for 8 h, followed by the addition of PLGA (1.6 g) and continued stirring to achieve a PLGA/nHA mixed solution. The resultant solutions were drawn into a syringe and electrospun using an electrospinning machine (Foshan Qingshi Co., Ltd., E02-001, China). The electrospinning parameters were kept constant and included an electrostatic field of 12 kV with the flow rate set to 1 mL h^-1^, a 20 G needle, a collector distance of 14 cm, a temperature of 25 °C ± 3 °C, and a relative humidity of 45% ± 5%. The collector rotation speed was set to 2,000 rpm for aligned fiber scaffolds and 400 rpm for random fiber scaffolds. Electrospinning was carried out for 2 h under the above parameters to yield four scaffold groups, namely, aligned PLGA/nHA fiber scaffolds (a-PLGA/nHA), random PLGA/nHA fiber scaffolds (r-PLGA/nHA), aligned PLGA fiber scaffolds (a-PLGA), and random PLGA fiber scaffolds (r-PLGA). Finally, the collected membranes underwent drying in a vacuum desiccator (Shanghai Boxun Industry, China) for 24 h and stored in a sealed environment.

### 2.4 Characterization

To examine the surface morphology of the samples and the nHA elemental distribution, the samples were imaged using a scanning electron microscopy (SEM, Thermos Scientific, Apreo 2C, Waltham, MA, United States) at an accelerating voltage of 10 kV. The elemental distribution of nHA was analyzed by energy-dispersive spectroscopy (EDS) mapping. For distribution, fiber diameter and orientation were measured from at least 50 randomly selected fibers at different locations, and fibers parallel to the y-axis were defined as having an orientation of 0°. The particle-size distribution of nHA powder was determined with a Laser Nanoparticle Size and Zeta Potential Analyzer (Brookhaven Instruments Corporation, 90Plus PALS, Holtsville, NY, United States). The crystalline structures of the samples were assessed with an X-ray diffractometer (Rigaku, Ultima IV, Tokyo, Japan). Functional groups were investigated using Fourier-transform infrared spectroscopy (FTIR, Nengpu Technology Co., Ltd., iCAN 9, Tianjin, China) over the range of 500–4,000 cm^-1^ at a resolution of 4 cm^-1^. Mechanical properties were measured under dry conditions using an electromechanical universal testing machine (MARK-10, FS05-2, Copigue, NY, United States). Thermal stability was assessed using a thermogravimetric analyzer (TGA, Mettler Toledo, TGA2, Zurich, Switzerland). The surface hydrophilicity of the membranes was analyzed using a contact angle goniometer (Krüss, DSA100, Hamburg, Germany). The degradation behavior of the samples was evaluated in PBS solution (pH = 7.45) at 37 °C. The release level of nHA was indirectly assessed by measuring the calcium ion release from different samples using a calcium ion detection kit.

### 2.5 Isolation and culture of hPDLSCs

Prior to initiation, the study protocol received approval from the Medical Ethics Committee of Guizhou Medical University (Approval No. 2022–17). The periodontal tissues were isolated from premolars or third molars with caries-free and healthy periodontal tissues that were extracted for orthodontic or impaction reasons. HPDLSCs were isolated from the collected tissues by 3 mg/mL collagenase type I and 4 mg/mL dispase II trypsin digestion within 1 h and primarily cultured at 37 °C in a humidified atmosphere of 5% CO_2_ (Li et al., 2015). Afterward, upon reaching 80% confluence, the cells were passaged using a trypsin solution and further expanded. The cells utilized in this study were from passages 3 to 5.

### 2.6 Cell seeding

Each group of electrospun fiber scaffolds was sectioned into 2 × 2 cm squares and placed in 12-well plates. The scaffolds underwent sterilization using 75% ethanol and UV irradiation, and were subsequently subjected to multiple phosphate-buffered saline (PBS) washes. Subsequently, hPDLSCs were seeded onto scaffolds at a density of 5 × 10^4^ cells per well for observation of cell morphology and osteogenic differentiation. The growth medium was replaced with osteogenic differentiation medium (formulation: α-MEM, 5% FBS, 1% penicillin-streptomycin, 10 nM dexamethasone, 50 μg/mL ascorbic acid, 10 mM β-glycerophosphate). Subsequent culture continued for 7 and 14 days, with medium changed every 2–3 days.

### 2.7 Morphological observation of hPDLSCs on the scaffolds

After culturing for 1 day, the scaffolds were washed with PBS and then fixed with 4% glutaraldehyde for 10 min. Cells were permeabilized with 0.5% Triton X-100 for 10 min and then blocked with 1% BSA for an additional 10 min. Subsequently, the cells on the substrates were stained with phalloidin-conjugated FITC to visualize F-actin (cytoskeleton) and with DAPI to label nuclei. The samples were then examined using a confocal laser-scanning microscope (CLSM, Olympus/Nikon A1, Tokyo, Japan).

SEM was also used to further investigate the morphological characteristics of cells cultured on the scaffolds. After culturing for 1 and 3 days, the scaffolds were rinsed with PBS and then fixed with 2.5% glutaraldehyde. After sequential dehydration in ethanol series and vacuum freeze-drying for 24 h, morphology of hPDLSCs on scaffolds was visualized by SEM.

### 2.8 Biocompatibility evaluation

Cell proliferation was evaluated with the CCK-8 assay. Each scaffold was sectioned into pieces measuring 0.6 × 0.6 cm squares, disinfected, and laid down at the bottom of 96-well plates. Subsequently, hPDLSCs were placed on the scaffolds to culture at the density of 5 × 10^3^ cells/well. On days 1, 3, and 5, a multifunctional microplate reader (Infinite F50, Tecan Group Ltd., Männedorf, Switzerland) was used to read the absorbance at 450 nm. L929 cells (a mouse fibroblast cell line) were seeded onto the material at a density of 1 × 10^4^ cells/well. After 1 day of culture, cells were stained with a live/dead staining kit for 30 min and then observed under an inverted fluorescence microscope (Leica DMi8, Leica Microsystems, Wetzlar, Hesse, Germany).

### 2.9 Quantitative real-time polymerase chain reaction (RT-qPCR)

HPDLSCs were seeded onto the scaffolds and subjected to osteogenic induction for 7 days. We extracted total RNA from samples using TRIzol reagent. Subsequently, the RNA was reverse transcribed into complementary DNA (cDNA). RT-qPCR was carried out using the ChamQ Universal SYBR qPCR Master Mix. The corresponding primer sequences for target genes are given in Table 1.

**TABLE 1:** Primer sequence of the osteogenic genes.

Primer name	Forward primer sequence (5‘−3’)	Reverse primer sequence (5‘−3’)
GAPDH	CAG​GAG​GCA​TTG​CTG​ATG​AT	GAAGGCTGGGGCTCATTT
OCN	GGT​GCA​GCC​TTT​GTG​TCC​AA	CCT​GAA​AGC​CGA​TGT​GGT​CA
RUNX2	CAG​ATG​GGA​CTG​TGG​TTA​CTG​T	AGG​TGA​AAC​TCT​TGC​CTC​GT
OPN	TTC​GCA​GAC​CTG​ACA​TCC​AG	ACG​GCT​GTC​CCA​ATC​AGA​AG

### 2.10 Evaluation of osteogenesis

Alkaline phosphatase (ALP) staining was used to evaluate the early stages of osteogenic differentiation of hPDLSCs on the scaffolds. After culturing the cells in osteogenic induction medium on the scaffolds for 7 days, the samples were fixed with 4% paraformaldehyde (Bioshap, China) for 30 min. The samples were incubated with ALP staining solution for 5–30 min. Subsequently, the stained specimens were observed under a stereomicroscope (Leica DMS1000, Wetzlar, Hesse, Germany).

To assay the late-stage osteogenic differentiation of cells on the scaffolds, after 14 days, cells were fixed *via* 30 min incubation in 4% paraformaldehyde. Then, they were subjected to alizarin red staining (ARS) for 10 min. Samples were rinsed with ddH_2_O, allowed to dry at room temperature, and subsequently imaged under a stereomicroscope. Furthermore, cellular mineralization was evaluated by SEM after culturing for 14 days.

### 2.11 YAP immunofluorescence staining

After 1 day of osteogenic induction using the same protocol, the culture medium was removed. Cells were fixed with 4% paraformaldehyde at room temperature for 30 min, followed by three washes with PBS (5 min each). Permeabilization was performed with 0.3% Triton X-100 for 15 min at room temperature, and samples were then blocked with 5% goat serum for 1 h. Subsequently, the cells were incubated with a primary antibody against YAP (1:100 dilution) overnight at 4 °C in the dark, rewarmed at room temperature for 30 min, and washed with PBST. A Cy3-conjugated secondary antibody (1:200 dilution) was applied and incubated for 1 h at room temperature in the dark. After washing with PBST, nuclei were stained with DAPI for 5 min. Finally, images were acquired using a laser scanning confocal microscope.

### 2.12 Statistical analysis

Data were shown as mean ± standard deviation (SD), and one-way analysis of variance (ANOVA) among groups was conducted. The following significance levels were designated: *P < 0.05, **P < 0.01, ***P < 0.001, and “ns” for non-significant results.

## 3 Results

### 3.1 Fabrication and characterization of nHA

**FIGURE 1:**
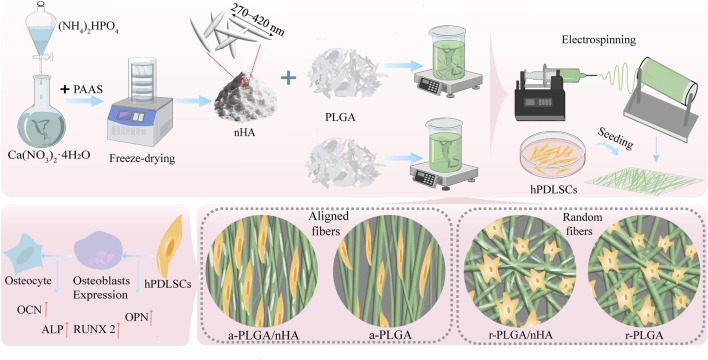
Diagram of the fabrication process of nHA and the scaffolds.

The fabrication processes are schematically illustrated in Figure 1. The nHA was synthesized through wet chemical precipitation, and the addition of PAAS significantly enhanced the dispersion of HA in the composite matrix.

**FIGURE 2:**
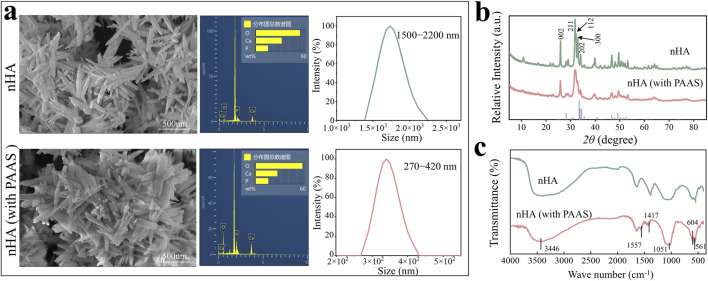
Evaluation results of nHA (with and without PAAS): **(a)** SEM images, elemental distribution and size distribution. **(b)** XRD patterns. **(c)** FTIR spectra.

As shown in Figure 2a, nHA samples with and without PAAS all demonstrated needle-type morphology, and EDS test indicated the same composition. Notably, PAAS incorporation significantly reduced nHA particle size from about 1,500 to 2,200 nm to 270–420 nm. In Figure 2b, PAAS incorporation preserved the nHA crystal phase but markedly reduced its crystallinity. The nHA (with and without PAAS) displayed characteristic peaks (002), (211), and (112). In comparison to nHA (without PAAS), the intensities of all diffraction peaks of nHA (with PAAS) were decreased. The functional groups of nHA were examined using FTIR spectroscopy (Figure 2c). A broad absorption peak near 3,602 cm^-1^ was primarily due to the −OH stretching vibration. The peak at 1,100 cm^-1^ was attributed to PO_4_^3-^ symmetric stretching, serving as a typical feature of nHA. Additionally, the absorption peaks identified at 560 cm^-1^ and 600 cm^-1^ were indicative of the symmetric stretching vibration modes of the PO_4_^3-^ group. Moreover, two minor peaks at 1,560 cm^-1^ and 1,420 cm^-1^ in the nHA (with PAAS) were attributed to the presence of PAAS.

### 3.2 Characterization of scaffolds

**FIGURE 3:**
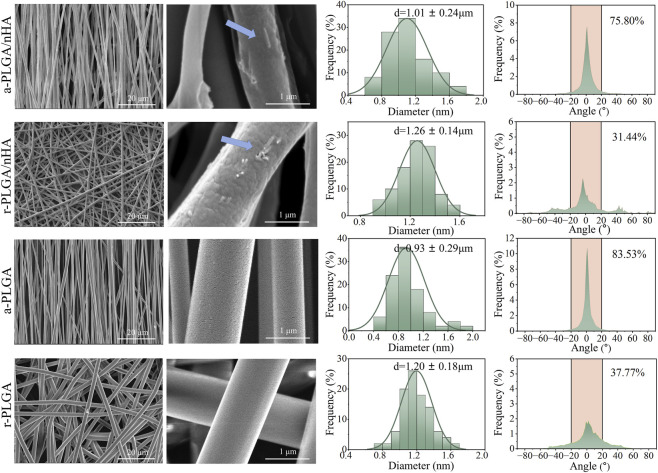
SEM images, fiber diameter distribution, and orientation distribution results of the scaffolds (Blue arrows indicated the nHA).

As illustrated in Figure 3, a-PLGA and r-PLGA fibers exhibited uniform, smooth surfaces devoid of bead formation. In the composite electrospun scaffolds (a-PLGA/nHA and r-PLGA/nHA), the nHA particles were uniformly dispersed and remained stably embedded within the PLGA nanofibers. Supplementary Figure S1 further demonstrated the uniform distribution of nHA within the fibers. From the quantitative analysis, we can see that the mean fiber diameter of composite scaffolds was a little bit higher than the pure PLGA one. In addition, a-PLGA/nHA and a-PLGA fiber scaffolds had a considerable orientation distribution in the set 0° ± 20°, which was 75.80% and 83.53% respectively. On the contrary, r-PLGA/nHA and r-PLGA fiber scaffolds showed an orientation of 31.44% and 37.77% respectively.

**FIGURE 4:**
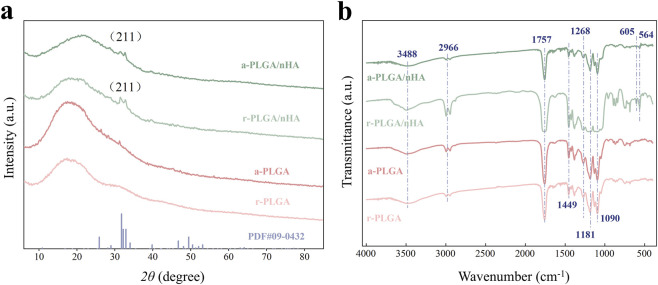
**(a)** XRD patterns and **(b)** FTIR spectra of the fiber scaffolds.

According to the XRD patterns in Figure 4a, a-PLGA/nHA and r-PLGA/nHA fiber scaffolds exhibited broad, diffuse peaks in the 2θ range of 10–25°, which were consistent with an amorphous structure of PLGA matrix. Notably, the characteristic diffraction peaks of PLGA in the composite scaffolds (a-PLGA/nHA and PLGA/nHA) were moderately attenuated, indicating that the incorporation of nHA disrupts the chain packing of PLGA and reduces its overall crystallinity. In addition, a-PLGA/nHA and r-PLGA/nHA fiber scaffolds exhibited distinct diffraction signals corresponding to those of pristine nHA (JCPDS No. 09–0,432), notably, the (211) reflection at 31.8°. It is worth noting that the intensity of this peak reduced in the a-PLGA/nHA compared to r-PLGA/nHA. It revealed that the oriented alignment of fibers may suppress the crystallographic development of the crystal faces of nHA.

The ester carbonyl group (C=O) of PLGA across all scaffolds consistently exhibited characteristic stretching vibration peaks at 1757 cm^-1^ (Figure 4b), indicating purely physical incorporation of nHA without chemical modification of PLGA. Additionally, primary characteristic peaks were attributed to PLGA, including the C−O−C group (1,090 cm^-1^, 1,181 cm^-1^), O−H (1,268 cm^-1^), C−H (1,449 cm^-1^), and methylene and methyl groups (2,800–3,300 cm^-1^). Notably, characteristic PO_4_^3-^ symmetric vibration peaks of nHA at 564 and 605 cm^-1^ were detected in a-PLGA/nHA and r-PLGA/nHA fiber scaffolds, confirming nHA incorporation. It was consistent with the result of the XRD analysis.

**FIGURE 5:**
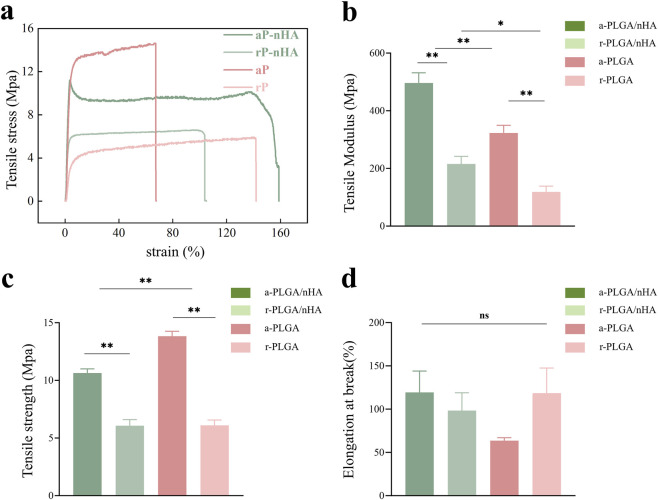
Mechanical properties tests of the scaffolds for **(a)** Stress-strain curves. **(b)** Tensile modulus histogram. **(c)** Tensile strength histogram. **(d)** Elongation at break histogram (*P < 0.05, **P < 0.01, and ns represents no significant difference).

To evaluate the mechanical properties of the scaffolds, a stress-strain analysis was carried out (Figure 5a), and the corresponding histograms of tensile modulus, tensile strength, and elongation at break were constructed accordingly (Figures 5b–d). Specifically, the tensile strength and tensile modulus of a-PLGA fiber scaffolds (13.83 MPa, 322.96 MPa) increased by 126.72% and 172.75%, respectively, compared with r-PLGA (6.10 MPa and 118.41 MPa). The incorporation of nHA significantly enhanced the tensile modulus of both aligned and random scaffolds (Figure 5b). Particularly, the tensile modulus of a-PLGA/nHA fiber scaffolds (496.06 MPa) was 53.60% higher than that of a-PLGA (322.96 MPa). Conversely, the tensile strength of scaffolds decreased upon the addition of nHA (Figure 5c). The a-PLGA/nHA fiber scaffolds declined 23.07% to 10.64 MPa compared with r-PLGA (6.10 MPa). We found a-PLGA/nHA fiber scaffolds exhibited higher elongation at break (119.28%) than a-PLGA (63.50%) (Figure 5d), indicating reduced brittleness. In contrast, the r-PLGA/nHA fiber scaffolds exhibited a decrease in elongation at break compared to the r-PLGA scaffolds, although the difference was not statistically significant. It may be attributable to non-uniform fiber thickness and interfacial bonding differences between aligned and random architectures.

**FIGURE 6:**
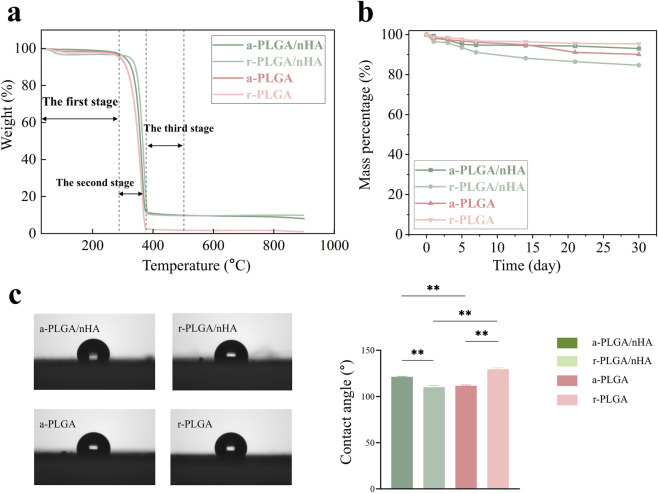
**(a)** TGA curves of the scaffolds. **(b)** Degradation curves of the scaffolds over 1 month. **(c)** Contact angle measurement images of the scaffolds and corresponding quantitative analysis (*P < 0.05, **P < 0.01).

Thermal stability and nHA loading of the scaffolds were quantitatively evaluated by TGA (Figure 6a), the initial mass loss (<4%) below 200 C was attributed to the evaporation of residual solvents. In the second stage (300–400 °C), the onset decomposition temperature of r-PLGA/nHA fiber scaffolds was 341 °C, higher than that of a-PLGA/nHA (327 °C), and both values exceeded that of a-PLGA and r-PLGA scaffolds (∼310 °C). The residual mass of a-PLGA and r-PLGA approaches zero at 600 °C. Whereas the composite scaffolds yielded a residual mass of ∼9%, in excellent agreement with the theoretical nHA content. As shown in Figure 6b, all sample groups maintained stability over an extended period (1 month). Calcium ion release from the r-PLGA/nHA scaffolds was minimal over 7 days and essentially negligible (Supplementary Table S1). Notably, the release from a-PLGA/nHA scaffolds was below the lower limit of detection of the assay kit. These findings further confirmed the stability of the materials. As shown in Figure 6c, a-PLGA scaffolds exhibited lower contact angles than r-PLGA scaffolds, indicating greater hydrophilicity. After nHA incorporation, the contact angle of r-PLGA/nHA scaffolds (109.88°) decreased significantly compared with r-PLGA (129.41°), reflecting enhanced hydrophilicity, whereas the contact angle of a-PLGA/nHA scaffolds increased relative to a-PLGA.

### 3.3 Morphology and proliferation of hPDLSCs on the scaffolds

**FIGURE 7:**
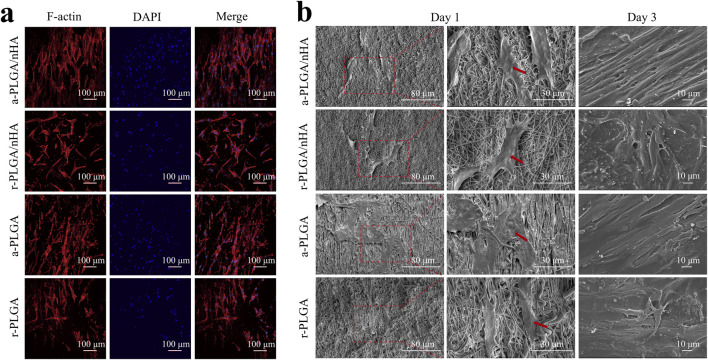
**(a)** CLSM images of hPDLSCs cultured on the scaffolds on day 1 (Nuclei were stained blue, F-actin was stained red.) **(b)** SEM images of hPDLSCs cultured on the scaffolds on day 1 and day 3 (Red arrows indicated the hPDLSCs).

**FIGURE 8:**
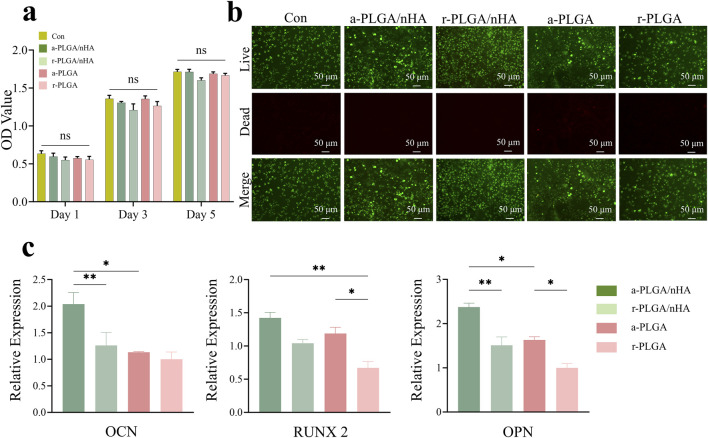
**(a)** CCK8 assay of hPDLSCs on the scaffolds. **(b)** Live/dead staining images of L929 cells cultured on the scaffolds for 1 day. **(c)** RT-qPCR expression of related mRNA (*P < 0.05 and **P < 0.01, and “ns” denoted a non-significant difference).

HPDLSCs exhibited markedly distinct growth morphological features on aligned *versus* random scaffolds. Specifically, the CSLM (Figure 7a) and SEM (Figure 7b) images revealed that hPDLSCs on the two aligned scaffolds elongated preferentially along the fiber direction, with well-organized F-actin cytoskeleton alignment, whereas cells on the random scaffolds adopted a polygonal morphology. At 3 days, SEM images further revealed these distinct growth characteristics of cells on aligned *versus* random scaffolds. CCK-8 assays (Figure 8a) showed that hPDLSCs proliferated over time on all scaffolds. Although the OD values of all groups were lower than those of the control, the differences were not statistically significant, suggesting that the materials did not exhibit significant cytotoxicity. Consistent with the data presented in Figure 8b, the fiber scaffolds from all groups demonstrated excellent biocompatibility.

### 3.4 Evaluation of hPDLSCs osteogenic differentiation potential on the scaffolds

Figure 8c showed that after 7 days, the aligned scaffolds (a-PLGA/nHA and a-PLGA) exhibited significantly higher levels of OCN, RUNX2, and OPN expression compared to random scaffolds (r-PLGA/nHA and r-PLGA). Notably, the mRNA levels of the three examined genes for a-PLGA/nHA scaffolds were all upregulated relative to the other scaffolds (*P* < 0.05).

**FIGURE 9:**
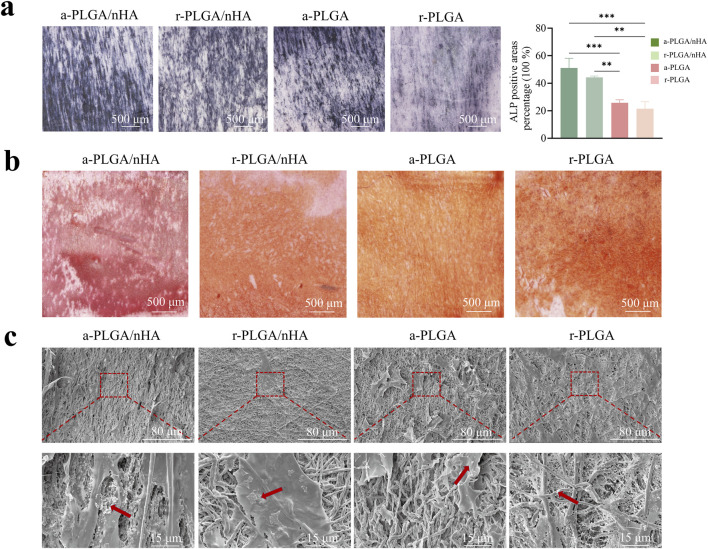
**(a)** ALP staining results and **(b)** ARS staining results of hPDLSCs cultured on the scaffolds. **(c)** SEM images of calcium in hPDLSCs cultured on the scaffolds for 14 days (Red arrows indicated the calcium ion deposition).

After 7 days, hPDLSCs began to exhibit ALP activity, as indicated by the varying staining intensities shown in Figure 9a. Cells cultured on aligned scaffolds demonstrated significantly higher ALP activity compared to those on random scaffolds. Meanwhile, the composite scaffolds exhibited more intense staining than their respective pure PLGA scaffolds, irrespective of fiber alignment. The semi-quantitative analysis of ALP in Figure 9a further verified that the a-PLGA/nHA fibrous scaffold exhibited the highest ALP activity. The ARS staining results (Figure 9b) showed that a-PLGA/nHA scaffolds exhibited stronger staining than the other three scaffolds. After 14 days of osteogenic induction, mineralized nodules could be seen in all scaffolds (Figure 9c).

**FIGURE 10:**
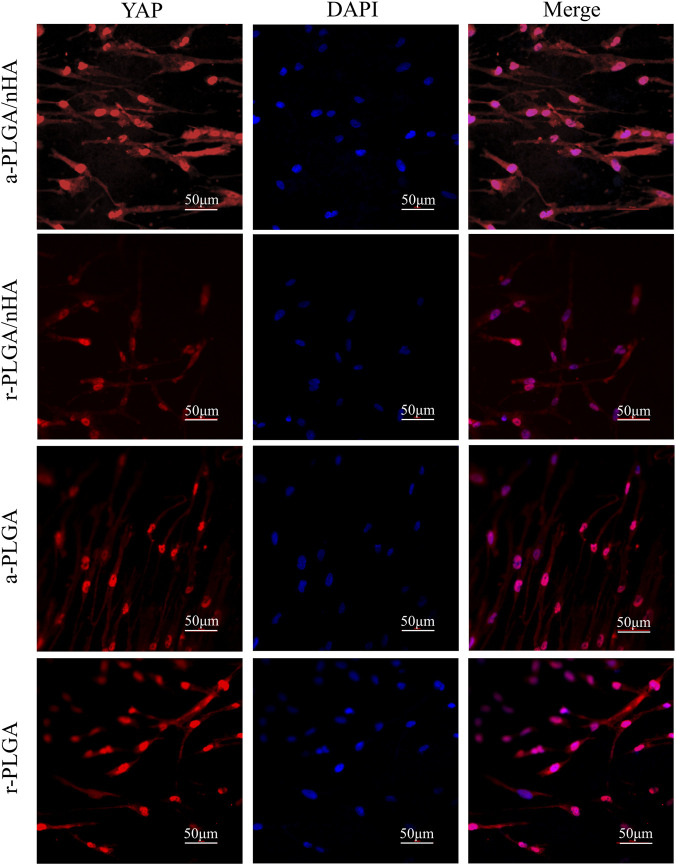
CLSM images of hPDLSCs cultured on the scaffolds on day 1 (YAP protein localization was stained red, nuclei were stained blue).

As shown in Figure 10, in the aligned groups (a-PLGA and a-PLGA/nHA), YAP protein (red fluorescence) was predominantly localized in the nucleus, exhibiting strong overlap with DAPI-stained nuclei (blue fluorescence). The fluorescence intensity in the nucleus was significantly higher than that in the cytoplasm, indicating a clear nuclear translocation. In contrast, in the random groups (r-PLGA and r-PLGA/nHA), only a small number of cells showed weak nuclear YAP signal, while in most cells YAP remained diffusely distributed in the cytoplasm. Notably, the a-PLGA/nHA group displayed the strongest nuclear red fluorescence intensity for YAP, demonstrating the most pronounced nuclear translocation.

## 4 Discussion

In this work, based on our previous study (Wang et al., 2015)and wet chemical precipitation method, aligned PLGA/nHA scaffolds with micro-/nanostructures were fabricated. This composite scaffold uniquely integrates the advantages of aligned fiber architecture, micro-/nanostructured surface topography, and nHA. This combination imparts superior osteogenic properties to the scaffold. It was concluded that aligned PLGA/nHA fiber scaffolds were able to cause hPDLSCs to differentiate in an osteogenic direction effectively. This work may provide preliminary guidance for scaffold design in alveolar bone tissue engineering applications.

The nHA, as a bioceramic material, is often combined with natural or synthetic polymers to make high-performance scaffolds for bone tissue engineering (Cakmak et al., 2020; Zaszczyńska et al., 2024; Mirică et al., 2021). The formation of nHA was reinforced with PAAS added as well for better dispersion in a polymer matrix, which improves the anti-aggregation properties of nHA (Deng et al., 2020; Tang et al., 2024). In addition, this surface-active agent also has the ability to change the surface charge of nHA, thus promoting cellular contact (Li et al., 2015). The results demonstrated that the incorporation of PAAS markedly reduced the particle size of nHA (Figure 2a). The nHA particles are dispersed evenly inside the PLGA fibers by PAAS adsorbing electrostatically on the surface of the particles (Figure 3) (Yan et al., 2018). High crystallinity is usually reflected in the XRD pattern as sharp and intense peaks (Figure 2b). However, nHA (with PAAS) exhibited diminished peak intensities and thus a lower degree of crystallinity. It may be attributed to the attenuation of diffraction intensity arising from the PAAS layer electrostatically anchored on the nHA surface, analogous to the peak suppression reported for PEG-modified nHA (Hajimirzaee et al., 2019; Hassan et al., 2022). FTIR spectra (Figure 2c) verified that the PO_4_^3-^ signature of nHA was retained after PAAS incorporation, confirming that the crystal structure of nHA remained intact (Hajimirzaee et al., 2019).

In tissue-engineering scaffolds, fiber alignment critically determines both mechanical performance and bioactivity. Aligned scaffolds (a-PLGA/nHA and a-PLGA) displayed significantly superior tensile strength and modulus over random scaffolds in Figure 5. Additionally, a-PLGA scaffolds exhibited significantly higher tensile strength but lower elongation at break than r-PLGA scaffolds, consistent with earlier observations (Fusaro et al., 2020; Trappmann et al., 2012). Although parallel-aligned fibers can effectively transmit tensile loads, their low nodal density makes them prone to stress concentration and microcrack formation. Once the local stress exceeds the critical threshold, cracks propagate catastrophically, ultimately leading to brittle failure of the scaffold (Li et al., 2020; Khoo et al., 2019). We found that incorporation of nHA markedly increased the tensile modulus of both aligned and random fiber scaffolds, indicating that an optimal content of nHA effectively reinforces material stiffness and resistance to deformation. However, the tensile strength of the aligned scaffold decreased. Combined with the analysis in Figure 4a, the characteristic PLGA peak intensity of a-PLGA/nHA scaffolds was significantly lower than that of the a-PLGA scaffolds. This reduction is likely attributable to nHA particles disrupting the stretch-induced orientation and crystallization of PLGA chains during electrospinning. Since the load is primarily transmitted through the oriented crystalline domains of the fibers (Naseem et al., 2021; Ding et al., 2022), this effect weakened the tensile strength of the aligned fibers. In contrast, the random fibers exhibited lower crystallinity and thus were less affected. Besides, the random architecture exhibited reduced elongation at break. These agglomerations act as stress concentrators, thereby compromising scaffold toughness (Deng et al., 2020; Hajimirzaee et al., 2019). Nevertheless, a-PLGA/nHA scaffolds exhibited superior mechanical properties.

Compared with the remaining two samples, a-PLGA/nHA and r-PLGA/nHA scaffolds exhibited elevated onset degradation temperatures, denoting superior thermal stability. Meanwhile, the hydrophilicity of the scaffold influences efficient cell adhesion, proliferation, and differentiation (Cai et al., 2020). Compared to r-PLGA scaffolds, a-PLGA scaffolds posed a significantly lower water contact angle (Figure 6c). This may be attributed to the more uniform surface-energy distribution that the well-aligned fiber surface provided, which facilitated the spreading and penetration of water molecules (Xie et al., 2021). With the incorporation of nHA, the water contact angle of r-PLGA/nHA scaffolds decreased significantly. We speculated that it was due to the intrinsic hydrophilicity of nHA, whose surface–OH groups hydrogen-bond with water molecules, thereby lowering the contact angle and modulating the surface free energy (Zaszczyńska et al., 2024). Conversely, the water contact angle of a-PLGA/nHA scaffolds increased, attributed to enhanced surface roughness induced by nHA, which impeded the spreading of water molecules within the scaffold (Ying et al., 2020). Nevertheless, a-PLGA/nHA fiber scaffolds exhibited favorable mechanical properties and thermal stability while concurrently enhancing hydrophilicity to fulfill the requirements for repairing alveolar bone defects.

Morphological changes of cells constitute the most immediate response to scaffold surface anisotropy (Downing et al., 2013). Satisfactory cell adhesion was observed on all scaffolds in the early phase, irrespective of the differences in diameter between aligned and random fibers (Figure 7). It indicated that fiber diameter did not exert a substantial influence on cell adhesion, similar to other studies (Xu et al., 2022; Yu et al., 2020). Additionally, on aligned scaffolds, F-actin cables were oriented parallel to the fibers, whereas random scaffolds produced stellate morphologies. As others reported, fibers affected cellular behavior through cytoskeletal dynamics (Dejob et al., 2021; Mosher et al., 2021). Aligned scaffolds promote cell orientation along the fiber direction and potentiate lineage-specific differentiation (Puhl et al., 2020; El Khatib et al., 2020). Moreover, scaffolds that present micro-/nanoscale topologies are enabled to accurately recapitulate the extracellular matrix (ECM) microenvironment (Verdugo-Avello et al., 2025). These hierarchical surface cues actively direct the reassembly and remodeling of the cytoskeletal network, enabling cells to maintain tensile homeostasis in three dimensions (Yang et al., 2025; Chen et al., 2021).

The expressions of the osteogenic transcription factors OCN, RUNX2, and OPN(Xie et al., 2021; Shang et al., 2021) were highest on a-PLGA/nHA scaffolds compared to the other three fibers (Figure 8c). Aligned fibers also upregulated ALP activity and calcium nodule deposition, with the a-PLGA/nHA scaffolds exhibiting the most significant mineralization (Figure 9). These data indicated that, a unique synergistic effect emerges between nHA and aligned PLGA fibers at the specific ratio. This combination results in significantly superior osteogenic outcomes compared to control groups containing a single component (pure PLGA) or a single structure (random fibers).

In this study, the aligned PLGA fibers were designed to mimic the collagen fiber arrangement of natural alveolar bone, with nHA uniformly dispersed within the fibers acting as the mineral component. This configuration created an organic-inorganic composite architecture that closely resembles the hierarchical structure of natural alveolar bone (Moten et al., 2022). The physical topology of the aligned fibers provides directional contact guidance for hPDLSCs, thereby promoting the organized alignment of the cytoskeleton along the fiber axis and inducing anisotropic cellular tension (Yang et al., 2025; Chen et al., 2021). In this study, the uniformly dispersed nHA facilitates sustained release of calcium and phosphate ions. More importantly, it functions as a “mechanical signal amplifier” by significantly enhancing cellular adhesion strength and spreading area on the fibers, thereby synergistically amplifying the internal cellular tension generated by the aligned fiber architecture. This enhanced cellular tension, together with the intrinsic chemical signals released by nHA, may collectively promote osteogenic differentiation through mechanisms potentially involving the YAP/TAZ signaling pathway. Notably, our immunofluorescence staining results showed that hPDLSCs cultured on PLGA/nHA fiber scaffolds exhibited marked nuclear translocation of YAP protein, whereas YAP was predominantly localized in the cytoplasm in the planar control group. This observation is highly consistent with the activation mechanism of the YAP/TAZ signaling pathway.

However, the osteogenic impact of fiber alignment on hPDLSCshas not reached a consensus yet. While our earlier study demonstrated that aligned PLGA scaffolds upregulate osteogenic markers at both gene and protein levels (Wang et al., 2015), recent reports have shown otherwise. Ren et al. observed lower ALP and OPN expression in hPDLSCs cultured on aligned fibers compared with random fibers (Ren et al., 2017), and Xu et al. reported that aligned architectures promoted periodontal-ligament rather than osteogenic differentiation (Xu et al., 2022). These differences may be due to the scaffold specific structural parameters which are very important for the process of differentiation. Substrate stiffness, for instance, has been shown to direct lineage commitment (D Angelo et al., 2019; Liu et al., 2018), with cells preferentially migrating to and proliferating on stiffer regions (D Angelo et al., 2019). Additionally, the interplay between mechanical cues and topographical features further modulates bone remodeling (Yang et al., 2025; Chen et al., 2021). All together indicate the complexity of scaffold Design of directing hPDLSCs differentiation.

To sum up, aligned PLGA/nHA composite scaffolds with a specific high mass ratio have been successfully prepared to partially mimic the micro-/nanostructure of alveolar bone. Then their physicochemical properties and effects on the osteogenic differentiation of hPDLSCs were observed. The results showed that the scaffolds exhibited enhanced mechanical strength, thermal stability, and hydrophilicity, and significantly promoted the adhesion, proliferation, and osteogenic differentiation of hPDLSCs. At this specific ratio, a unique synergistic effect between nHA and aligned PLGA fibers was observed, demonstrating superior performance in promoting the osteogenic differentiation of hPDLSCs. These findings provide a basis for the optimization of electrospinning parameters in the design of periodontal scaffolds.

## Data Availability

The original contributions presented in the study are included in the article/Supplementary Material, further inquiries can be directed to the corresponding authors.
