# Supplementary material for: Aligned PLGA/nHA fiber scaffolds for enhancing osteogenic differentiation of human periodontal ligament stem cells
Source: Front Bioeng Biotechnol. 2026 Jun 11;14:1802077. doi: 10.3389/fbioe.2026.1802077 (PMC13294402; doi:10.3389/fbioe.2026.1802077)
Supplement: Supplementary file 1 [file DataSheet1.PDF]

Appendix

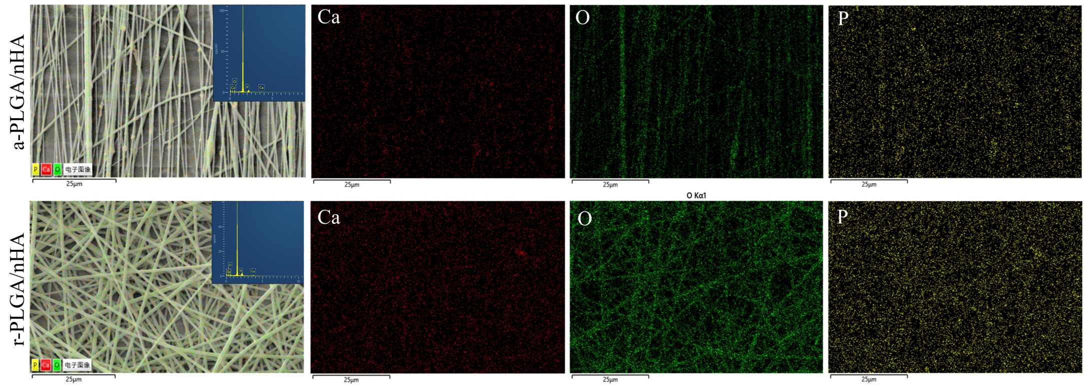

Figure S1. Energy dispersive spectroscopy (EDS) mapping of the composite fiber scaffold.

Table S1. Concentrations of Ca^{2+} ions in PBS solution samples obtained by four fiber samples in 1 ml PBS solution (originally without Ca^{2+} ions) at 37 °C for different times (1~7 days).

Samples	1 day	3 days	7 days
a-PLGA/nHA	<0.01 mM	<0.01 mM	<0.01 mM
r-PLGA/nHA	0.02 mM	0.03 mM	0.03 mM
a-PLGA	<0.01 mM	<0.01 mM	<0.01 mM
r-PLGA	<0.01 mM	<0.01 mM	<0.01 mM
